# Obesity Is Associated with Lower Coronary Microvascular Density

**DOI:** 10.1371/journal.pone.0081798

**Published:** 2013-11-29

**Authors:** Duncan J. Campbell, Jithendra B. Somaratne, David L. Prior, Michael Yii, James F. Kenny, Andrew E. Newcomb, Darren J. Kelly, Mary Jane Black

**Affiliations:** 1 St. Vincent’s Institute of Medical Research, Fitzroy, Australia; 2 Department of Medicine, The University of Melbourne, St. Vincent's Health, Fitzroy, Australia; 3 Department of Cardiology, St. Vincent's Health, Fitzroy, Australia; 4 Department of Surgery, University of Melbourne, St. Vincent's Health, Fitzroy, Australia; 5 Department of Cardiothoracic Surgery, St. Vincent's Health, Fitzroy, Australia; 6 Department of Anatomy and Developmental Biology, Monash University, Clayton, Australia; Scientific Directorate, Bambino Hospital, Italy

## Abstract

**Background:**

Obesity is associated with diastolic dysfunction, lower maximal myocardial blood flow, impaired myocardial metabolism and increased risk of heart failure. We examined the association between obesity, left ventricular filling pressure and myocardial structure.

**Methods:**

We performed histological analysis of non-ischemic myocardium from 57 patients (46 men and 11 women) undergoing coronary artery bypass graft surgery who did not have previous cardiac surgery, myocardial infarction, heart failure, atrial fibrillation or loop diuretic therapy.

**Results:**

Non-obese (body mass index, BMI, ≤30 kg/m^2^, n=33) and obese patients (BMI >30 kg/m^2^, n=24) did not differ with respect to myocardial total, interstitial or perivascular fibrosis, arteriolar dimensions, or cardiomyocyte width. Obese patients had lower capillary length density (1145±239, mean±SD, vs. 1371±333 mm/mm^3^, *P*=0.007) and higher diffusion radius (16.9±1.5 vs. 15.6±2.0 μm, *P*=0.012), in comparison with non-obese patients. However, the diffusion radius/cardiomyocyte width ratio of obese patients (0.73±0.11 μm/μm) was not significantly different from that of non-obese patients (0.71±0.11 μm/μm), suggesting that differences in cardiomyocyte width explained in part the differences in capillary length density and diffusion radius between non-obese and obese patients. Increased BMI was associated with increased pulmonary capillary wedge pressure (PCWP, *P*<0.0001), and lower capillary length density was associated with both increased BMI (*P*=0.043) and increased PCWP (*P*=0.016).

**Conclusions:**

Obesity and its accompanying increase in left ventricular filling pressure were associated with lower coronary microvascular density, which may contribute to the lower maximal myocardial blood flow, impaired myocardial metabolism, diastolic dysfunction and higher risk of heart failure in obese individuals.

## Introduction

The increasing prevalence of obesity is a major health concern. Increased body mass index (BMI) has a well-established association with diastolic dysfunction and risk of heart failure, and diastolic dysfunction is a precursor to heart failure [1-3]. The mechanisms involved in the progression of increased BMI to diastolic dysfunction and heart failure remain uncertain [4]. Increased BMI is a risk factor for hypertension, diabetes and dyslipidemia, all of which augment the risk of ischemic heart disease, and hypertension and diabetes independently increase the risk of heart failure [5]. In addition, elevated BMI is associated with increased left ventricular (LV) mass [5,6], and altered LV remodeling [6]; however, the association of BMI with diastolic dysfunction is independent of age, hypertension, diabetes and LV mass [1,2]. Other mechanisms by which BMI may impact on diastolic function and risk of heart failure include altered myocardial structure, neurohormonal activation and altered myocardial metabolism [4,7-9].

Animal models show obesity to be associated with cardiac hypertrophy and alterations in myocardial structure and coronary microvasculature [10-16]. Humans with increased BMI have impaired maximal myocardial blood flow [17,18], but the mechanism of impaired myocardial blood flow of obese subjects is unknown. One possible mechanism is lower capillary density, which may contribute to impaired cardiomyocyte metabolism through mismatch of myocardial oxygen supply and demand [19], leading to myocardial decompensation and heart failure [20].

To investigate the hypothesis that obesity and its accompanying diastolic dysfunction are associated with alteration in myocardial structure we performed histological analysis of non-ischemic LV myocardial biopsies from patients without heart failure or previous myocardial infarction who were undergoing coronary artery bypass graft surgery. We previously reported that neither age, diabetes nor the metabolic syndrome was associated with altered myocardial total or interstitial fibrosis, cardiomyocyte width, capillary length density, diffusion radius or arteriolar dimensions in men from this patient population, although men with diabetes and the metabolic syndrome had lower perivascular fibrosis [21,22]. In the present study we show that, in contrast to the effects of age, diabetes and the metabolic syndrome, obesity and its accompanying increase in LV filling pressure were associated with lower coronary microvascular density that may contribute to the impaired maximal myocardial blood flow, diastolic dysfunction and increased risk of heart failure in obese individuals.

## Methods

The St. Vincent's Health Human Research Ethics Committee approved this research and all patients gave written informed consent.

### Patients

Details of the Cardiac Tissue Bank have been previously described [23]. From the Tissue Bank we selected all of 57 patients (46 men and 11 women) having coronary artery bypass graft surgery alone; none had previous cardiac surgery, heart failure or atrial fibrillation, had received loop diuretic therapy or had evidence of previous myocardial infarction. Absence of previous myocardial infarction was established from the clinical history, electrocardiogram and troponin measurements, and was confirmed by inspection of the ventriculogram, transthoracic and transesophageal echocardiography and examination of the heart at surgery. All patients had normal or near-normal LV systolic function as assessed by pre-operative transthoracic echocardiography and ventriculogram, with LV ejection fraction ≥50%. A partial-thickness wedge-shaped biopsy was taken during surgery, immediately after cardioplegia, from a region of the lateral wall of the LV near the base of the heart, between the territories of the left anterior descending and circumflex arteries, that was free of any macroscopic pathology and without evidence of ischemia or wall motion abnormality on pre-operative or intra-operative imaging studies.

Each patient had a Swan-Ganz catheter inserted before surgery that provided a measure of pulmonary artery pressure, pulmonary capillary wedge pressure (PCWP) and cardiac output that were recorded immediately after induction of anesthesia. PCWP was measured at end-expiration by temporarily disconnecting the patient from the ventilator at the time of measurement, as previously shown to provide an accurate measure of left ventricular end-diastolic pressure [24].

Fifteen patients had type 2 diabetes mellitus, another 27 had the metabolic syndrome and 15 had neither condition. The metabolic syndrome was defined according to the International Diabetes Federation [25]. For patients in whom abdominal circumference was not measured, based on the relationship between abdominal circumference and BMI [26], those with BMI>25 kg/m^2^ were considered to exceed the abdominal circumference threshold for the metabolic syndrome. A patient had diabetes if a history of diabetes was evident from use of glucose-lowering medications and/or insulin or if the fasting plasma glucose was ≥7 mmol/L [27].

### Biochemistry

Blood hemoglobin and hemoglobin A1c and plasma creatinine were measured as part of the routine pre-surgery workup. All other variables were measured on fasting blood collected before induction of anesthesia on the day of surgery. Estimated glomerular filtration rate (eGFR) was calculated from the Modification of Diet in Renal Disease formula [28]. Insulin resistance (HOMA2-IR), insulin sensitivity (HOMA2-%S) and ß-cell function (HOMA2-%B) were calculated using the HOMA calculator version 2.2 [29]. Amino-terminal-pro-B-type natriuretic peptide (NT-proBNP) was measured by electrochemiluminescence immunoassay using an Elecsys instrument (Roche Diagnostics, Basel, Switzerland).

### Histological analysis

Details of tissue collection, fixation and histology have been previously described [23]. All histological analyses were performed blind to patient identity and characteristics. Picrosirius red-stained 4 μm sections of paraffin-embedded tissue were analyzed for total, interstitial and perivascular fibrosis and arteriolar dimensions by quantitative morphometry of digitized images of the whole myocardial section (Aperio Technologies, Inc., CA) as previously described [23]. Myocardial total fibrosis was calculated using the positive pixel count algorithm as the area of collagen staining expressed as a percentage of the total myocardial tissue area, after excluding the pericardium, whereas interstitial fibrosis was calculated as described for total fibrosis, with exclusion of perivascular fibrosis.

Arterioles were identified by the presence of a layer of media and immunohistochemical staining for elastin showed the blood vessels were relaxed. The tissue was immersion fixed and the arterioles were usually oval in shape because of deformation and/or because they were cut at an oblique angle. We did not attempt to analyze arterioles in longitudinal section, and only arterioles in approximate cross-section or oblique-section were analyzed for perivascular fibrosis. Perivascular fibrosis ratio was calculated as the ratio of the area of perivascular fibrosis to the total vessel area (area of vessel wall plus lumen). Arteriolar wall area/circumference ratio was calculated for arterioles with average diameters of 20-80 μm, which represented 86% of all arterioles counted.

Cardiomyocyte width, determined on 4 μm sections of paraffin-embedded tissue (one section per patient) stained for reticulin, was the mean of >100 measurements for each section of the shortest diameter of cardiomyocyte profiles containing a nucleus. Capillary length density, which is the length of capillaries per unit volume of tissue, and diffusion radius, were determined by analysis of 4 μm sections of paraffin-embedded tissue (one section per patient) immunostained for CD31 (mouse anti-human CD31 monoclonal antibody, Dako Denmark A/S, Glostrup, Denmark) using standard stereological techniques as previously described [23].

### Statistical methods

Data are presented as mean±SD for normally distributed variables and as median with 25th and 75th percentiles for variables that were not normally distributed. The normality of continuous data was verified with the Kolmogorov-Smirnov test and variables with a positively skewed distribution were log transformed before analysis. Categorical variables are expressed as number (%). Differences between groups were tested with *t*-test for continuous variables and χ^2^ or Fisher's exact tests for discrete variables. Regression analysis was performed using the method of least squares and correlations were estimated using Pearson correlation coefficients. All tests were two-tailed. Calculations were performed using Statview statistical software (SAS Institute Inc) and a two-sided *P* value of <0.05 was considered to indicate statistical significance.

## Results

### Study patients

**Figure 1:**
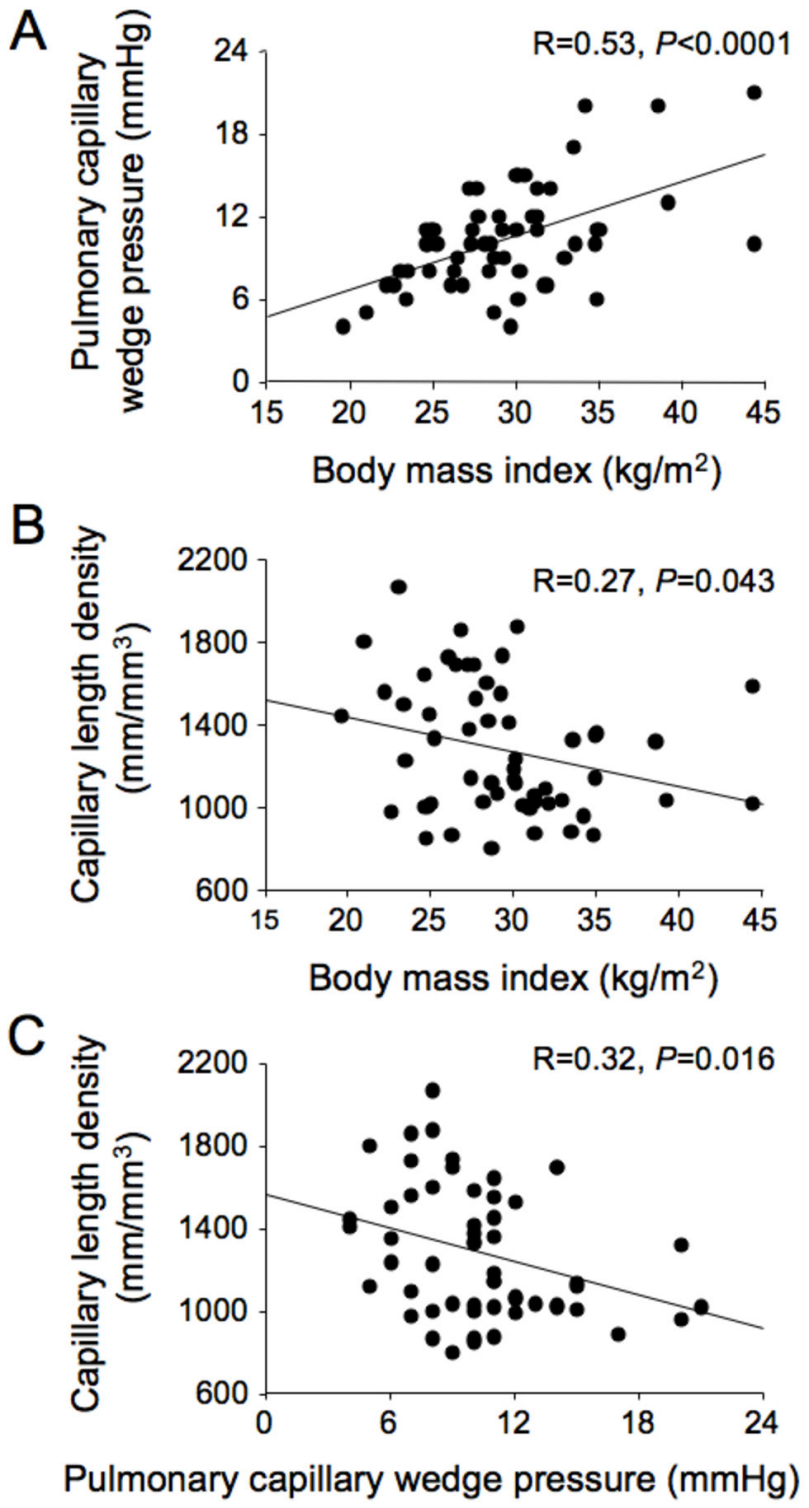
Correlations between pulmonary capillary wedge pressure, body mass index and capillary length density. Pulmonary capillary wedge pressure was correlated with body mass index (A); moreover, capillary length density was correlated with body mass index (B) and pulmonary capillary wedge pressure (C) in 57 coronary artery bypass graft surgery patients.

The clinical, biochemical and hemodynamic characteristics of the study patients are shown in Table 1. Non-obese and obese patients did not differ with respect to age, gender, or extent of coronary artery disease, as measured by numbers of coronary arteries with stenoses, occluded vessels, coronary collaterals, bypass grafts, previous percutaneous transluminal coronary angioplasty or wall motion abnormalities. Body weight, BMI, and body surface area (BSA) were higher in obese patients whereas height was lower in obese than non-obese patients. A greater proportion of obese patients had diabetes or the metabolic syndrome and a history of hypertension, but blood pressures did not differ between the two groups at pre-admission or during surgery. Obese patients had higher plasma triglyceride and insulin levels and insulin resistance, but the two groups did not differ with respect to plasma levels of NT-proBNP and C-reactive protein or eGFR. There were no differences in medication use between non-obese and obese patients except for higher use of thiazide or indapamide therapy by obese patients. Obese patients had increased PCWP, central venous and pulmonary artery pressures, and PCWP was correlated with BMI (Figure 1). 

**Table 1:** Clinical, biochemical and hemodynamic characteristics of coronary artery bypass graft surgery patients with BMI ≤30 kg/m^2^ and >30 kg/m^2^.

Characteristic		BMI ≤30 kg/m^2^ (n=33)	BMI >30 kg/m^2^ (n=24)	P
Age, years		64±10	63±10	0.70
Women, n (%)		4 (12%)	7 (29%)	0.17
Left main stenosis >50%, n (%)		14 (42%)	12 (50%)	0.60
One vessel stenosis >70%, n (%)		7 (21%)	7 (29%)	0.54
Two vessel stenosis >70%, n (%)		17 (52%)	11 (46%)	0.79
Three vessel stenosis >70%, n (%)		8 (24%)	5 (21%)	1.0
Patients with occluded coronary artery, n (%)		12 (36%)	8 (33%)	1.0
Coronary collaterals, Rentrop grade 2 or 3, n (%)		16 (48%)	11 (46%)	1.0
Wall motion abnormality		4 (12%)	2 (8%)	1.0
Previous percutaneous transluminal coronary angioplasty, n (%)		4 (12%)	3 (13%)	1.0
Coronary artery conduits/patient, n		3±1	3±1	0.71
Body weight (kg)		79±12	96±12	<0.0001
Height (cm)		174±9	168±10	0.025
Body mass index (kg/m^2^)		26±3	34±4	<0.0001
BSA (m^2^)		1.9±0.2	2.0±0.2	0.022
Clinical risk factors				
	Diabetes, n (%)	6 (18%)	9 (38%)	0.13
	Metabolic syndrome (non-diabetic), n (%)	13 (39%)	14 (58%)	0.19
	Diabetes or metabolic syndrome, n (%)	19 (58%)	23 (96%)	0.002
	Pre-admission SBP (mmHg)	132±15	133±15	0.87
	Pre-admission DBP (mmHg)	75±8	76±7	0.77
	Previous hypertension, n (%)	19 (58%)	20 (83%)	0.048
	Use of tobacco, ever, n (%)	16 (48%)	17 (71%)	0.11
	Fasting plasma total cholesterol (mmol/L)	3.7±1.2	3.3±0.7	0.08
	Fasting plasma LDL cholesterol (mmol/L)	2.3±1.0	1.8±0.7	0.07
	Fasting plasma HDL cholesterol (mmol/L)	0.98±0.20	0.92±0.23	0.34
	Fasting plasma triglyceride (mmol/L)	1.2 (1.0-1.7)	1.7 (1.3-2.7)	0.008
	Fasting plasma glucose (mmol/L)	6.1±1.3	6.2±1.3	0.71
	Fasting plasma insulin (pmol/L)	43 (29-66)	86 (61-129)	0.001
	ß cell function from HOMA2-%B	58 (46-80)	81 (69-117)	0.005
	Insulin sensitivity from HOMA2-%S	122 (80-191)	60 (42-88)	0.0008
	Insulin resistance from HOMA2-IR	0.8 (0.5-1.2)	1.7 (1.2-2.4)	0.0006
	Plasma NT-proBNP (pmol/L)	10 (4-27)	11 (5-23)	0.61
	eGFR (mL/min per 1.73 m^2^)	73±14	68±15	0.13
	C-reactive protein (mg/L)	1.1 (0.6-4.3)	2.1 (1.1-4.8)	0.40
Medications				
	ACE inhibitor therapy, n (%)	14 (42%)	17 (71%)	0.06
	ARB therapy, n (%)	10 (30%)	5 (21%)	0.55
	ACEI and/or ARB therapy, (%)	24 (73%)	20 (83%)	0.52
	Statin therapy, n (%)	27 (82%)	21 (88%)	0.72
	Aspirin therapy, n (%)	28 (85%)	24 (100%)	0.07
	Calcium antagonist therapy, n (%)	7 (21%)	8 (33%)	0.37
	ß-blocker therapy, n (%)	23 (70%)	20 (83%)	0.35
	Long-acting nitrate therapy, n (%)	8 (24%)	6 (25%)	1.0
	Thiazide or indapamide therapy, n (%)	5 (15%)	10 (42%)	0.035
Intra-operative hemodynamics immediately post induction of anesthesia				
	Central venous pressure (mmHg)	8±4	10±4	0.015
	Pulmonary capillary wedge pressure (mmHg)	9±3	12±4	0.003
	Mean pulmonary artery pressure (mmHg)	15±4	19±5	0.002
	Mean arterial pressure (mmHg)	74±11	76±14	0.51
	Cardiac index (L/min/m^2^)	2.6±0.8	2.4±0.5	0.22

Continuous data are expressed as mean±SD or median (interquartile range) for variables with skewed distribution, and categorical variables are expressed as number (%). One non-obese and one obese patient had left main stenosis without other vessel stenosis >70%. Coronary collaterals were scored according to Rentrop et al. [46]. ACE, angiotensin converting enzyme; ARB, angiotensin receptor blocker; BSA, body surface area; eGFR, estimated glomerular filtration rate calculated using the Modification of Diet in Renal Disease study equation [28]; HDL, high density lipoprotein; HOMA, Homeostasis Model Assessment calculator version 2.2 [29]; LDL, low density lipoprotein; NT-proBNP, amino-terminal-pro-B-type natriuretic peptide. Comparison of parameters for patients with BMI ≤30 kg/m^2^ and >30 kg/m^2^ were performed using *t*-test for continuous variables and χ^2^ or Fisher's exact tests for discrete variables.

### Association of obesity with myocardial structure

**Figure 2:**
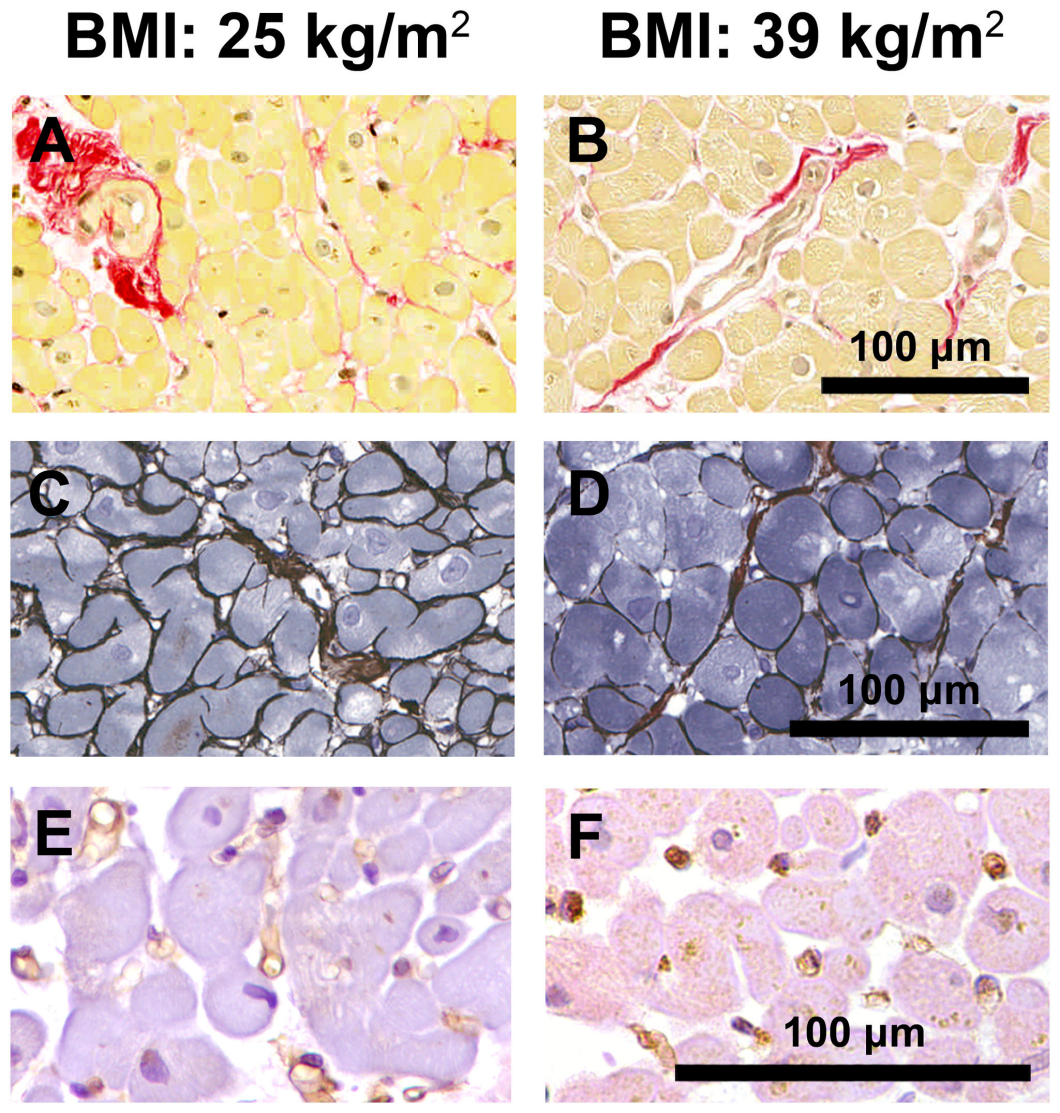
Picrosirius-red staining of collagen, reticulin staining of cardiomyocyte membranes, and CD31 immunostaining of capillaries. Representative sections of left ventricular biopsies from a non-obese male (BMI: 25 kg/m^2^) and an obese male (BMI: 39 kg/m^2^) coronary artery bypass graft surgery patient stained with picrosirius-red demonstrating interstitial and perivascular fibrosis (stained red) and arteriolar dimensions (A, B), reticulin stain demonstrating cardiomyocyte membranes (C, D), and immunostained for CD31 demonstrating capillaries (E, F).

All biopsies were taken from a region of the LV wall without evidence of ischemia or wall motion abnormality and none of the biopsies showed evidence of ischemia (Figure 2). There were no differences between non-obese and obese patients with respect to total, interstitial or perivascular fibrosis, arteriolar dimensions, or cardiomyocyte width (Table 2). However, obese patients had lower capillary length density and increased diffusion radius, in comparison with non-obese patients, accompanied by a reduction in arteriolar density that was of borderline statistical significance. Although the higher cardiomyocyte width of obese patients was not statistically significantly different from that of non-obese patients, the diffusion radius/cardiomyocyte width ratios of obese and non-obese patients were not significantly different (Table 2). Lower capillary length density, but not myocardial fibrosis, arteriolar dimensions or cardiomyocyte width, was associated with both increased BMI and increased PCWP (Figure 1).

**Table 2:** Histology of left ventricular biopsies of coronary artery bypass graft surgery patients with BMI ≤30 kg/m^2^ and >30 kg/m^2^.

Characteristic	BMI ≤30 kg/m^2^ (n=33)	BMI >30 kg/m^2^ (n=24)	P
Myocardium area per section (mm^2^)	4.1±2.2	4.3±2.4	0.70
Total fibrosis (%)	2.0±0.9	1.9±0.9	0.68
Interstitial fibrosis (%)	1.5±0.7	1.4±0.7	0.77
Perivascular fibrosis ratio	1.9±1.2	1.9±1.0	0.86
Arterioles/mm^2^ myocardium area	1.2±0.7	0.9±0.3	0.07
Mean arteriolar diameter, all arterioles (μm)	39±14	40±16	0.74
Arteriolar wall area/circumference ratio (μm^2^/μm)	5.5±1.8	5.0±1.5	0.33
Capillary length density (mm/mm^3^)	1371±333	1146±239	0.007
Diffusion radius (μm)	15.6±2.0	16.9±1.5	0.012
Diffusion radius/BSA ratio (μm/m^2^)	8.1±1.1	8.3±1.0	0.49
Cardiomyocyte width (μm)	22.1±2.5	23.4±4.5	0.16
Cardiomyocyte width/BSA ratio (μm/m^2^)	11.5±1.4	11.4±1.8	0.89
Diffusion radius/cardiomyocyte width ratio (μm/μm)	0.71±0.11	0.73±0.11	0.56

Data are expressed as mean±SD. BSA, body surface area. Myocardium area per section excludes epicardium. We did not attempt to analyze arterioles in longitudinal section, and only arterioles in approximate cross-section or oblique section with diameters (average of maximum and minimum diameter of each arteriole) of 12-151 μm were counted for estimation of arteriolar density and analyzed for perivascular fibrosis. Arteriolar wall area/circumference ratio was calculated for arterioles with diameters of 20-80 μm. Arteriolar wall area/circumference ratio and capillary length density and diffusion radius were measured for 33 non-obese and 23 obese patients. Comparison of parameters for patients with BMI ≤30 kg/m^2^ and >30 kg/m^2^ were performed using *t*-test for continuous variables and χ^2^ or Fisher's exact tests for discrete variables.

When men and women were analyzed separately, capillary length density of the 17 obese men was less than that of the 29 non-obese men (*P*=0.035); however, the lower capillary length density of the 7 obese women did not achieve statistical significance in comparison with the 4 non-obese women (*P*=0.10).

Although obese patients were more likely to have hypertension, capillary length density was not associated with a history of hypertension or blood pressure at pre-admission or during surgery. In this population of 56 men and women, in comparison with patients without diabetes or the metabolic syndrome, analysis of variance showed that patients with diabetes (*P*=0.036), but not the metabolic syndrome, had lower capillary length density. However, in regression analysis for the whole patient population, capillary length density was not associated with fasting plasma glucose, log fasting plasma insulin or log plasma triglyceride levels, or ß cell function (log HOMA2-%B), insulin sensitivity (log HOMA2-%S) or insulin resistance (log HOMA2-IR).

## Discussion

We confirmed the well-established association between BMI and diastolic dysfunction [1-3], and we report for the first time that increased BMI and its accompanying increase in LV filling pressure were associated with lower coronary microvascular density and increased diffusion radius. These findings provide a structural basis for the lower maximal myocardial blood flow of obese individuals [17,18]. In addition, our finding that cardiac fibrosis was similar for non-obese and obese patients indicates that fibrosis was not a contributor to the diastolic dysfunction of obese individuals. Cardiomyocyte size is a critical determinant of capillary length density in the adult because, in contrast to children, adults do not show a compensatory angiogenic response to cardiomyocyte hypertrophy [30,31]. Although the difference in cardiomyocyte width between non-obese and obese patients was not statistically significant, their similar diffusion radius/cardiomyocyte width ratios suggests that differences in cardiomyocyte width explains in part the differences in capillary length density and diffusion radius between non-obese and obese patients.

Among all organs, the heart is unique in that oxygen extraction is constantly close to maximal and the importance of the capillary bed to the ischemic vulnerability of the myocardium is well recognised [32-34]. Reduction in capillary length density in obesity may contribute to impaired cardiomyocyte metabolism and ATP production through mismatch of myocardial oxygen demand and supply [19], leading to myocardial decompensation and heart failure [20]. As diastole is more susceptible to ATP shortage than systole, impaired ATP production would initially manifest as diastolic dysfunction [7]. Cardiomyocytes of obese individuals may be particularly susceptible to ischemia because of the increased oxygen requirements for oxidation of free fatty acids and the lesser ability of cardiomyocytes to switch to utilization of glucose as a substrate [9]. A recent case control study showed that bariatric surgery reduced the risk of heart failure in obese patients with diabetes [35], but future studies are required to determine whether reduction in obesity increases an individual's coronary microvascular density. Our finding of lower coronary microvascular density of obese patients is consistent with the report of a lower skin capillary density in both normotensive and hypertensive patients with severe obesity; however, skin capillary density was not normalized after pronounced weight loss following bariatric surgery [36].

A reduction in the number or density of microvessels has been reported in both clinical and experimental hypertension and diabetes [37], raising the possibility that these conditions may account for the lower coronary microvascular density in obese subjects in our study. Evidence against a role for hypertension was the lack of association between capillary length density and history of hypertension or blood pressure at pre-admission or during surgery. Blockers of the renin angiotensin system improve microvascular structure in experimental hypertension and in hypertensive patients [37], which may explain the lack of association between history of hypertension and coronary microvascular density in our study, given the high proportion of patients receiving therapy with blockers of the renin angiotensin system. Therapy with blockers of the renin angiotensin system may also explain recent reports of normal capillary density in skin of patients with hypertension and/or diabetes [36,38].

Previous studies of the mechanism of the association between increased BMI, diastolic dysfunction and the risk of heart failure have had difficulty separating the effects of BMI from the effects of the metabolic syndrome, impaired insulin sensitivity and cardiac hypertrophy [8,39,40], and some studies suggested that the metabolic syndrome, and not increased BMI per se, is associated with increased risk of heart failure [41,42]. We previously reported that PCWP was increased in men with diabetes and the metabolic syndrome [21], but we found that neither diabetes nor the metabolic syndrome was associated with alteration in capillary length density [21]. However, in the present analysis of a larger cohort that included both men and women, capillary length density was lower in patients with diabetes, but not in patients with the metabolic syndrome alone, in comparison with patients without either condition. Further evidence that the lower coronary microvascular density of obese patients was independent of the metabolic syndrome was the lack of association between capillary length density and parameters of insulin sensitivity.

Our study highlights both similarities and differences between the effects of obesity on the myocardium of humans and animals. Animal models show obesity to be associated with cardiac hypertrophy [10-14], and increased cardiomyocyte size in some studies [10,11,13], but not in others [15]. Myocardial capillary density is reported to be either lower [10], similar to [11,12,14], or higher in obese than non-obese animals [12,14]. Although we found increased BMI was associated with lower capillary length density, we did not observe the association between BMI and arteriolar wall thickness that was reported in obese rats [16]. In contrast to reports of increased cardiac fibrosis in animal models of obesity [13-16], we found no association between BMI and myocardial fibrosis. In one autopsy study of obese individuals, 7 were reported to have mild and 3 had moderate cardiac fibrosis [43], but the contribution of comorbidities to cardiac pathology in this autopsy study is unknown. Our finding of no relationship between BMI or PCWP and cardiac fibrosis indicates that fibrosis was not a contributor to the diastolic dysfunction of obese individuals and is in contrast to reports of echocardiographic evidence and plasma fibrosis markers suggestive of cardiac fibrosis in obesity [44,45].

Our study had a number of limitations. The sample size was limited by the need for myocardial biopsies from each patient and our study was therefore restricted to univariable analyses. Another limitation was the inherent selection bias caused by the sampling of patients presenting for coronary artery bypass graft surgery, and it is not known whether our findings apply to patients with less extensive coronary artery disease. However, patients with coronary artery disease were an important group to study because of the high prevalence of coronary artery disease in the community, and our previous studies showed that the presence of coronary artery disease per se does not influence myocardial fibrosis, cardiomyocyte width, capillary length density or arteriolar dimensions [34]. To avoid the effect of coronary stenoses on myocardial structure and the microvasculature we took particular care to collect biopsies from the same epicardial region of the LV myocardium without evidence of ischemia or wall motion abnormality that was proximal to significant flow-limiting coronary stenoses and collaterals. However, it cannot be guaranteed that the biopsies were from healthy and comparable parts of myocardium of different patients, as subclinical perfusion disturbances might not be obvious, and another limitation of this approach is that we do not know if the data obtained apply to other regions of the myocardium. Nevertheless, these potential sources of variability in myocardial histology would have biased our results toward the null hypothesis.

In summary, we showed that increased BMI and percent body fat and their accompanying increase in LV filling pressure were associated with lower coronary microvascular density, but not with alteration in myocardial fibrosis or arteriolar dimensions. Lower coronary microvascular density may contribute to the lower maximal myocardial blood flow, impaired myocardial metabolism, diastolic dysfunction and increased risk of heart failure risk in obese individuals.

## References

[1] Russo C, Jin Z, Homma S, Rundek T, Elkind MS et al. (2011) Effect of obesity and overweight on left ventricular diastolic function: a community-based study in an elderly cohort. J Am Coll Cardiol 57: 1368-1374. doi:10.1016/S0735-1097(11)61368-5. PubMed: 21414533. 21414533 PMC3077126

[2] Cil H, Bulur S, Türker Y, Kaya A, Alemdar R et al. (2012) Impact of body mass index on left ventricular diastolic dysfunction. Echocardiography 29: 647-651. doi:10.1111/j.1540-8175.2012.01688.x. PubMed: 22486526. 22486526

[3] Kane GC, Karon BL, Mahoney DW, Redfield MM, Roger VL et al. (2011) Progression of left ventricular diastolic dysfunction and risk of heart failure. JAMA 306: 856-863. doi:10.1001/jama.2011.1201. PubMed: 21862747. 21862747 PMC3269764

[4] Horwich TB, Fonarow GC (2010) Glucose, obesity, metabolic syndrome, and diabetes relevance to incidence of heart failure. J Am Coll Cardiol 55: 283-293. doi:10.1016/j.jacc.2009.07.029. PubMed: 20117431. 20117431 PMC2834416

[5] Bombelli M, Facchetti R, Sega R, Carugo S, Fodri D et al. (2011) Impact of body mass index and waist circumference on the long-term risk of diabetes mellitus, hypertension, and cardiac organ damage. Hypertension 58: 1029-1035. doi:10.1161/HYPERTENSIONAHA.111.175125. PubMed: 22025375. 22025375

[6] Turkbey EB, McClelland RL, Kronmal RA, Burke GL, Bild DE et al. (2010) The impact of obesity on the left ventricle: the Multi-Ethnic Study of Atherosclerosis (MESA). JACC Cardiovasc Imaging 3: 266-274. doi:10.1016/j.jcmg.2009.10.012. PubMed: 20223423. 20223423 PMC3037096

[7] Rider OJ, Cox P, Tyler D, Clarke K, Neubauer S (2013) Myocardial substrate metabolism in obesity. Int J Obes, 37: 972–9. doi:10.1038/ijo.2012.170. PubMed: 23069666. 23069666

[8] Niemann B, Chen Y, Teschner M, Li L, Silber RE et al. (2011) Obesity induces signs of premature cardiac aging in younger patients: the role of mitochondria. J Am Coll Cardiol 57: 577-585. doi:10.1016/S0735-1097(11)60577-9. PubMed: 21272749. 21272749

[9] Szczepaniak LS, Victor RG, Orci L, Unger RH (2007) Forgotten but not gone: the rediscovery of fatty heart, the most common unrecognized disease in America. Circ Res 101: 759-767. doi:10.1161/CIRCRESAHA.107.160457. PubMed: 17932333. 17932333

[10] Toblli JE, Cao G, DeRosa G, Di Gennaro F, Forcada P (2004) Angiotensin-converting enzyme inhibition and angiogenesis in myocardium of obese Zucker rats. Am J Hypertens 17: 172-180. doi:10.1016/j.amjhyper.2004.03.452. PubMed: 14751661. 14751661

[11] Gruber C, Kohlstedt K, Loot AE, Fleming I, Kummer W et al. (2012) Stereological characterization of left ventricular cardiomyocytes, capillaries, and innervation in the nondiabetic, obese mouse. Cardiovasc Pathol 21: 346-354. doi:10.1016/j.carpath.2011.11.003. PubMed: 22197049. 22197049

[12] Sugawara T, Fujii S, Zaman AK, Goto D, Furumoto T et al. (2003) Coronary capillary network remodeling and hypofibrinolysis in aged obese diabetic rats: implications for increased myocardial vulnerability to ischemia. Mol Cell Biochem 248: 165-170. doi:10.1023/A:1024196504666. PubMed: 12870669. 12870669

[13] Qin F, Siwik DA, Luptak I, Hou X, Wang L, et al. (2012) The polyphenols resveratrol and S17834 prevent the structural and functional sequelae of diet-induced metabolic heart disease in mice. Circulation 125: 1757-1764, S1751-1756 22388319 10.1161/CIRCULATIONAHA.111.067801 PMC3354628

[14] Li ZL, Woollard JR, Ebrahimi B, Crane JA, Jordan KL et al. (2012) Transition from obesity to metabolic syndrome is associated with altered myocardial autophagy and apoptosis. Arterioscler Thromb Vasc Biol 32: 1132-1141. doi:10.1161/ATVBAHA.111.244061. PubMed: 22383702. 22383702 PMC3331917

[15] Leopoldo AS, Sugizaki MM, Lima-Leopoldo AP, do Nascimento AF, Luvizotto Rde A et al. (2010) Cardiac remodeling in a rat model of diet-induced obesity. Can J Cardiol 26: 423-429. doi:10.1016/S0828-282X(10)70440-2. PubMed: 20931095. 20931095 PMC2954535

[16] Burlá AK, Lobato NS, Fortes ZB, Oigman W, Neves MF (2013) Cardiac fibrosis and vascular remodeling are attenuated by metformin in obese rats. Int J Cardiol, 165: 483–487. doi:10.1016/j.ijcard.2011.09.012. PubMed: 21945710. 21945710

[17] Schindler TH, Cardenas J, Prior JO, Facta AD, Kreissl MC et al. (2006) Relationship between increasing body weight, insulin resistance, inflammation, adipocytokine leptin, and coronary circulatory function. J Am Coll Cardiol 47: 1188-1195. doi:10.1016/j.jacc.2005.10.062. PubMed: 16545651. 16545651

[18] Quercioli A, Pataky Z, Montecucco F, Carballo S, Thomas A et al. (2012) Coronary vasomotor control in obesity and morbid obesity: contrasting flow responses with endocannabinoids, leptin, and inflammation. JACC Cardiovasc Imaging 5: 805-815. doi:10.1016/j.jcmg.2012.01.020. PubMed: 22897994. 22897994

[19] Oka T, Komuro I (2008) Molecular mechanisms underlying the transition of cardiac hypertrophy to heart failure. Circ J 72 Suppl A: A13-A16. doi:10.1253/circj.CJ-08-0481. PubMed: 18772527. 18772527

[20] Walsh K, Shiojima I (2007) Cardiac growth and angiogenesis coordinated by intertissue interactions. J Clin Invest 117: 3176-3179. doi:10.1172/JCI34126. PubMed: 17975662. 17975662 PMC2045631

[21] Campbell DJ, Somaratne JB, Jenkins AJ, Prior DL, Yii M et al. (2011) Impact of type 2 diabetes and the metabolic syndrome on myocardial structure and microvasculature of men with coronary artery disease. Cardiovasc Diabetol 10: 80. doi:10.1186/1475-2840-10-80. PubMed: 21929744. 21929744 PMC3182888

[22] Campbell DJ, Somaratne JB, Jenkins AJ, Prior DL, Yii M et al. (2012) Diastolic dysfunction of aging is independent of myocardial structure but associated with plasma advanced glycation end-product levels. PLOS ONE 7: e49813. doi:10.1371/journal.pone.0049813. PubMed: 23189164. 23189164 PMC3506639

[23] Campbell DJ, Somaratne JB, Jenkins AJ, Prior DL, Yii M et al. (2011) Differences in myocardial structure and coronary microvasculature between men and women with coronary artery disease. Hypertension 57: 186-192. doi:10.1161/HYPERTENSIONAHA.110.165043. PubMed: 21135353. 21135353

[24] Ryan JJ, Rich JD, Thiruvoipati T, Swamy R, Kim GH et al. (2012) Current practice for determining pulmonary capillary wedge pressure predisposes to serious errors in the classification of patients with pulmonary hypertension. Am Heart J 163: 589-594. doi:10.1016/j.ahj.2012.01.024. PubMed: 22520524. 22520524

[25] Alberti KG, Zimmet P, Shaw J (2006) Metabolic syndrome--a new world-wide definition. A Consensus Statement from the International Diabetes Federation. Diabet Med 23: 469-480. doi:10.1007/BF02706751. PubMed: 16681555. 16681555

[26] Zhu S, Heshka S, Wang Z, Shen W, Allison DB et al. (2004) Combination of BMI and waist circumference for identifying cardiovascular risk factors in whites. Obes Res 12: 633-645. doi:10.1038/oby.2004.73. PubMed: 15090631. 15090631

[27] The Expert Committee on the Diagnosis and Classification of Diabetes Mellitus (2003) Report of the expert committee on the diagnosis and classification of diabetes mellitus. Diabetes Care 26 Suppl 1: S5-S20. doi:10.2337/diacare.26.2007.S5. PubMed: 12502614. 12502614

[28] Levey AS, Bosch JP, Lewis JB, Greene T, Rogers N et al. (1999) A more accurate method to estimate glomerular filtration rate from serum creatinine: a new prediction equation. Modification of Diet in Renal Disease Study Group. Ann Intern Med 130: 461-470. doi:10.7326/0003-4819-130-6-199903160-00002. PubMed: 10075613. 10075613

[29] Wallace TM, Levy JC, Matthews DR (2004) Use and abuse of HOMA modeling. Diabetes Care 27: 1487-1495. doi:10.2337/diacare.27.6.1487. PubMed: 15161807. 15161807

[30] Rakusan K, Flanagan MF, Geva T, Southern J, Van Praagh R (1992) Morphometry of human coronary capillaries during normal growth and the effect of age in left ventricular pressure-overload hypertrophy. Circulation 86: 38-46. doi:10.1161/01.CIR.86.1.38. PubMed: 1535573. 1535573

[31] Tomanek RJ (1992) Age as a modulator of coronary capillary angiogenesis. Circulation 86: 320-321. doi:10.1161/01.CIR.86.1.320. PubMed: 1617782. 1617782

[32] Messer JV, Wagman RJ, Levine HJ, Neill WA, Krasnow N et al. (1962) Patterns of human myocardial oxygen extraction during rest and exercise. J Clin Invest 41: 725-742. doi:10.1172/JCI104531. PubMed: 14472965. 14472965 PMC290976

[33] Anversa P, Sonnenblick EH (1990) Ischemic cardiomyopathy: pathophysiologic mechanisms. Prog Cardiovasc Dis 33: 49-70. doi:10.1016/0033-0620(90)90039-5. PubMed: 2142312. 2142312

[34] Campbell DJ, Somaratne JB, Jenkins AJ, Prior DL, Yii M et al. (2012) Reduced microvascular density in non-ischaemic myocardium of patients with recent non-ST-segment-elevation myocardial infarction. Int J Cardiol. doi:10.1016/j.ijcard.2012.03.075. 22459379

[35] Johnson BL, Blackhurst DW, Latham BB, Cull DL, Bour ES et al. (2013) Bariatric surgery Is associated with a reduction in major macrovascular and microvascular complications in moderately to severely obese patients with type 2 diabetes mellitus. J Am Coll Surg 216: 545-556. doi:10.1016/j.jamcollsurg.2012.12.019. PubMed: 23391591. 23391591

[36] De Ciuceis C, Rossini C, Porteri E, La Boria E, Corbellini C et al. (2013) Circulating endothelial progenitor cells, microvascular density and fibrosis in obesity before and after bariatric surgery. Blood Press 22: 165-172. doi:10.3109/08037051.2012.749584. PubMed: 23286244. 23286244

[37] Levy BI, Schiffrin EL, Mourad JJ, Agostini D, Vicaut E et al. (2008) Impaired tissue perfusion: a pathology common to hypertension, obesity, and diabetes mellitus. Circulation 118: 968-976. doi:10.1161/CIRCULATIONAHA.107.763730. PubMed: 18725503. 18725503

[38] Aellen J, Dabiri A, Heim A, Liaudet L, Burnier M et al. (2012) Preserved capillary density of dorsal finger skin in treated hypertensive patients with or without type 2 diabetes. Microcirculation 19: 554-562. doi:10.1111/j.1549-8719.2012.00188.x. PubMed: 22578093. 22578093

[39] Abel ED (2011) Obesity stresses cardiac mitochondria even when you are young. J Am Coll Cardiol 57: 586-589. doi:10.1016/S0735-1097(11)60586-X. PubMed: 21272750. 21272750

[40] Peterson LR, Herrero P, Schechtman KB, Racette SB, Waggoner AD et al. (2004) Effect of obesity and insulin resistance on myocardial substrate metabolism and efficiency in young women. Circulation 109: 2191-2196. doi:10.1161/01.CIR.0000127959.28627.F8. PubMed: 15123530. 15123530

[41] Ingelsson E, Sundström J, Arnlöv J, Zethelius B, Lind L (2005) Insulin resistance and risk of congestive heart failure. JAMA 294: 334-341. doi:10.1001/jama.294.3.334. PubMed: 16030278. 16030278

[42] Voulgari C, Tentolouris N, Dilaveris P, Tousoulis D, Katsilambros N et al. (2011) Increased heart failure risk in normal-weight people with metabolic syndrome compared with metabolically healthy obese individuals. J Am Coll Cardiol 58: 1343-1350. doi:10.1016/j.jacc.2011.04.047. PubMed: 21920263. 21920263

[43] Ahmed Q, Chung-Park M, Tomashefski JF Jr. (1997) Cardiopulmonary pathology in patients with sleep apnea/obesity hypoventilation syndrome. Hum Pathol 28: 264-269. doi:10.1016/S0046-8177(97)90122-2. PubMed: 9042788. 9042788

[44] Wong CY, O'Moore-Sullivan T, Leano R, Byrne N, Beller E et al. (2004) Alterations of left ventricular myocardial characteristics associated with obesity. Circulation 110: 3081-3087. doi:10.1161/01.CIR.0000147184.13872.0F. PubMed: 15520317. 15520317

[45] Kosmala W, Jedrzejuk D, Derzhko R, Przewlocka-Kosmala M, Mysiak A et al. (2012) Left ventricular function impairment in patients with normal-weight obesity: contribution of abdominal fat deposition, profibrotic state, reduced insulin sensitivity, and proinflammatory activation. Circ Cardiovasc Imaging 5: 349-356. doi:10.1161/CIRCIMAGING.111.969956. PubMed: 22407472. 22407472

[46] Rentrop KP, Cohen M, Blanke H, Phillips RA (1985) Changes in collateral channel filling immediately after controlled coronary artery occlusion by an angioplasty balloon in human subjects. J Am Coll Cardiol 5: 587-592. doi:10.1016/S0735-1097(85)80380-6. PubMed: 3156171. 3156171

